# Many Reviews Are Systematic but Some Are More Transparent and Completely Reported than Others

**DOI:** 10.1371/journal.pmed.0040147

**Published:** 2007-03-27

**Authors:** The PLoS Medicine Editors

## Abstract

The implications for PLoS Medicine and other journals of new research, published in this issue, showing that there are major variations in the reporting quality of systematic reviews.

The past decade has seen the establishment of the systematic review (SR) as one of the cornerstones of evidence-based medicine. The value of SRs to researchers, practitioners, and policy makers is well established; when done well, they are considered the highest level of evidence for medical decision making. Potentially, they are also a resource for patients seeking to make sense of the relative value of different treatments.

It is important to distinguish SRs from the traditional, narrative reviews also often published in medical journals. A helpful definition of a SR, which also clarifies the difference between an SR and meta-analysis, comes from the Web site (http://www.cochrane.org/resources/glossary.htm) of the Cochrane Collaboration (named after Archie Cochrane, a British medical researcher), an international organization that publishes rigorous SRs evaluating the effectiveness of a wide range of health care interventions. Their definition of a SR is this: “A review of a clearly formulated question that uses systematic and explicit methods to identify, select, and critically appraise relevant research, and to collect and analyze data from the studies that are included in the review. Statistical methods (meta-analysis) may or may not be used to analyze and summarize the results of the included studies.”

The increasing number of new SRs being published (currently estimated as being around 2,500 per year [1]) have become an essential part of the biomedical literature. But SRs that are of low quality or out of date have the potential to mislead, and selective publication of SRs that support particular agendas—or failure to publish those with “undesirable” results—could undermine the literature's reliability in the same way that biased publication of primary research can. How rigorously are these reviews being performed? How consistent are they in reporting their methods and their results?

In this issue we publish an article [1] by David Moher and colleagues that examines both the “epidemiological aspects” of published SRs and their “reporting characteristics.” It is not the first time SR quality has been examined, but this study is different in that it does not focus on a particular area of health care, one type of intervention, or a sample of journals, but on all SRs indexed on PubMed within a specified period of time—one month, November 2004. The authors included both SRs published in the Cochrane Database of Systematic Reviews (CDSR) and those published elsewhere. Although such a limited period can only be a snapshot, this detailed assessment of 300 reviews revealed several points of interest.

There were clear differences in the quality of reporting between Cochrane and non-Cochrane SRs. Many reviews included no mention of a pre-specified protocol by which the review was conducted. Although a protocol is required of all Cochrane reviews, only 11% of non-Cochrane reviews had one. This lack of a protocol—which delineates the search strategy, inclusion criteria, and the plan for the analysis that will be conducted—naturally leads to concerns about methodological rigor in the assessment of the study question.

It is Cochrane policy that published reviews should be regularly updated. One-third of the CDSR reviews in Moher's sample were in fact updates. However, updating is uncommon elsewhere; in the Moher sample, only 2% of non-Cochrane reviews were updates. Related to this issue is the observation that outside of the Cochrane Collaboration none of the reviews were registered with a central body. Hence, it would be hard to locate and access updates even if they were done.

The quality of reporting in many of the SRs was disappointing. Despite the guidelines of the Cochrane Collaboration and the QUOROM (Quality of Reporting of Meta-Analyses) initiative [2], important items were frequently missing, again mostly from non-Cochrane reviews. For example, only two-thirds of all reviews mentioned the years searched; an equal number gave information on quality assessment of studies included, and fewer than a quarter gave an indication of having assessed for publication bias. There was a lack of detail regarding eligibility criteria; many reviews did not make clear whether they were restricted by study type or whether data came only from published studies. Only three-quarters of the reviews dealt with potential harms (in addition to benefits) of the intervention under review.

Perhaps of most concern, the authors noted: “Strong evidence of outcome reporting bias was recently reported within clinical trials. Our results suggest that some aspect of selective outcome reporting bias might also exist within non-Cochrane reviews. Only about one-quarter of them reported a primary outcome, of which half report statistical significance in favour of this outcome (versus 14.4% for Cochrane reviews).”

Collectively, given the importance accorded to SRs, these findings are worrying. While length limitations imposed by most journals (but not by CDSR) may contribute to incomplete reporting, it seems more likely that many authors are not fully aware of the tools that are available to help them report SRs. Reporting all the items on the QUOROM checklist is neither onerous nor excessively lengthy; unfortunately many journals, especially the smaller subspecialty journals in which many SRs are published, have yet to adopt these reporting guidelines. For example, as Moher and colleagues point out, less than 7% overall (and, surprisingly, only 4% of Cochrane reviews) used a QUOROM flowchart to illustrate the stages of doing the systematic review.

PLoS is committed to publishing high-quality systematic reviews, and the online format of our journals allows flexibility in the length of the research articles we publish. However, the number of research articles that *PLoS Medicine* can accept is limited, so we are not able to publish every well-conducted SR that comes our way. We will restrict ourselves to publishing those that we believe have findings that represent an important advance that will interest our general medical audience.

PLoS's new journal, *PLoS ONE* is, however, able to publish many more papers than *PLoS Medicine* can. *PLoS ONE* is committed to publishing any SR that is conducted, described, and interpreted well, and it welcomes the submission of SRs across all areas of health care. Moreover, *PLoS ONE* can also publish updates of SRs, provided that the update itself contains sufficient detail for a reader who cannot access the original report to understand what was done. If the original report is freely available, the update can be linked electronically to the original publication. We require that that all SRs submitted to us include a protocol and QUOROM checklist (or any updated version of this checklist) submitted as supplementary files. These items will be made available to peer reviewers, and published with the paper. In order to help authors report SRs systematically, we now also provide an optional template that follows the current QUOROM checklist. This template will be updated as needed in future.

But no one journal or publisher can single-handedly address the issues involved here. Moher and colleagues call for the registration of SRs in the same manner as is now required for clinical trials: it would then, at least, be possible to track those that are performed. This is an interesting proposal, and would certainly help in the linking of SRs to updates. We would welcome opinions on this proposal.

Despite the best efforts of the Cochrane Collaboration and the QUOROM group, there is still a long way to go before readers can assume that all SRs are conducted and reported in a way that reliably provides an accurate synthesis of the evidence available.

The QUOROM group is responding to this situation. The group met toward the end of 2006 and has made major revisions to its guidelines. Additionally, to facilitate a better understanding and dissemination of the guidelines, the group has embarked on developing an explanation and elaboration document similar to that used to create the corresponding CONSORT (Consolidated Standards of Reporting Trials; for reporting of clinical trials) and STARD (Standards for Reporting of Diagnostic Accuracy; for reporting of diagnostic studies) documents. It is anticipated that both documents will be submitted for publication consideration around the middle of this year. The group will also be changing its name from the much misspelled “QUOROM” to PRISMA (Preferred Reporting Items for Systematic Reviews and Meta-Analyses).

We hope these developments will go some way to maximizing the dependability of systematic reviews and justifying their position as the gold standard of evidence in health care.

## Abbreviations

CDSR: Cochrane Database of Systematic Reviews
SR: systematic review
